# Standard precautions for preventing Tuberculosis and HIV: Compliance of Eswatini university student nurses

**DOI:** 10.1371/journal.pone.0261944

**Published:** 2021-12-30

**Authors:** Ncobile Sidzandza Victoria Gina, Melitah Molatelo Rasweswe, Miriam Mmamphamo Moagi

**Affiliations:** 1 Faculty of Health Sciences, Department of Nursing Science, University of Pretoria, Pretoria, South Africa; 2 Nursing department, Mahikeng Campus, North West University, Potchefstroom, South Africa; Jordan University of Science and Technology, JORDAN

## Abstract

Tuberculosis and Human Immunodeficiency Virus are among the top ten causes of death globally. To prevent the spread of these infections, health workers and student nurses should comply to infection prevention and control measures called standard precautions. The aim of this study is to assess compliance of Eswatini university student nurses regarding standard precautions for preventing Tuberculosis and Human Immunodeficiency Virus. A non-experimental quantitative approach was used to conduct a survey on all senior student nurses of Eswatini University using questionnaires. IBM SPSS Statistics version 26 software was used to analyse the data. Results from this study showed that out of the 105 student nurses who were asked only 51.4% (n = 54) said they always used personal protective equipment. However, they did comply well on disposing sharps as 92.4% (n = 97) reported that they always used designated containers. There is a need for close supervision of student nurses in the clinical area. The researcher recommends that clinical facilitator should always accompany student nurses in the clinical area and that preceptors should be exempted from other nursing duties when there are student nurses in the hospitals so that they can mentor the students.

## 1. Introduction and background

Healthcare workers, including student nurses face a lot of challenges including exposure to Tuberculosis (TB) and Human Immunodeficiency Virus (HIV) as they provide healthcare services. TB and HIV are among the top ten causes of death globally, 35 million people have died of HIV so far [1, 2] and in 2017 1.6 million people died of TB. Globally there were 36.9 million people living with HIV in 2017 and 6.7 million people were reported to the national TB program and the World Health Organization (WHO) to be infected with TB [1, 2]. One of the most essential measures used to control the spread of TB and HIV in the healthcare setting is the application of Standard Precautions. Standard precautions are a group of infection control safety measures used to reduce the risk of micro-organisms from both recognized and unrecognized sources of infections in health facilities [3].

Student nurses’ forms part of the healthcare team and should therefore comply to Standard Precautions to protect themselves and the patients under their care. However, many studies globally have reported lack of compliance to standard precautions [4]. One study showed that among the 50 student nurses 20% had a satisfactory level of performance of standard precautions [5]. The authors continue to argue that 66% of the students did not perform the handling and disposal of sharps as per the guideline. Seventy percent of students did not cover eyes and mouth during procedures which carries risk of splashing body fluids and 50% did not wash hands after removing gloves. This shows that compliance to standard precautions is still a challenge for a lot of health workers. Healthcare providers have a greater risk of exposure to HIV by needle sticks or cuts, and getting blood or other body fluids in their eyes or mouths, and blood or other body fluids on their skin when it is chapped, scraped or affected by skin inflammations [6].

Students are often more at risk for exposures because they are learning new skills and techniques that they are not confident performing [7]. Student nurses are persons studying towards a nursing qualification at a university or college or any private institution accredited by the South African Nursing Council (SANC) and Council of Higher Education [8]. Student nurses are exposed to HIV through needle pricks, blood and body fluids splashes and TB during consultations and monitoring of vital signs of coughing patients [9].

In Eswatini, many people are infected with HIV, the prevalence rate being at 31% among persons between ages 18–49 years [10]. Among the countries with the highest burden of TB, Eswatini has the highest estimated incidence of TB which is 398 cases per 100,000 population and the largest number of HIV-associated TB cases with 70% of TB patients estimated to be HIV-positive [11]. The high prevalence of TB and HIV rate in Eswatini places student nurses even in more danger of being infected during their practice in the clinical area. Low compliance rate to standard precautions among student nurses in the clinical area can lead to transmission of infectious diseases such as TB and HIV and have negative effects on the recruitment and training of nurses in the country. While a lot have been said about the compliance of student nurses to standard precautions in other countries, such studies have never been done in Eswatini.

Hence, it is important to assess student nurses’ compliance with standard precautions for preventing Tuberculosis and HIV infections to enable training institutions to design more effective strategies to improve compliance in the clinical area.

## 2. Materials and methods

### 2.1 The research setting

The study was carried out at the University of Eswatini (UNESWA) in Eswatini, a small land-locked country located in the southern part of Africa. The University of Eswatini (UNESWA) Faculty of Health Sciences, is located in the Hhohho region at Mbabane, the capital town of Eswatini. By virtue of being the largest and oldest tertiary institution in the country, the University of Eswatini was ideal for this study for its larger population of student nurses.

### 2.2 Research design

The researchers used a non-experimental quantitative descriptive research design when assessing compliance of UNESWA student nurses to standard precautions for preventing TB and HIV. A total population sampling method was used because the size of the population of interest was small. The total number of these senior nursing students in the academic year 2019/2020 was 144. There were 58 third years, 64 fourth year and 22 Fifth year and were all included in the study. Students in year three, four and year five were selected due to the fact that they have sufficient knowledge and experience with TB and HIV prevention. After writing the research proposal the researchers sought approval from the ethics committee of the University of Pretoria, Faculty of Health Sciences, then the Eswatini Health and Human Research Review Board and the UNESWA. The researchers were granted permission to conduct the study by these bodies. With these approvals, data was then collected on the student nurses who voluntarily consented to be part of the study. The data was collected between July and September 2020. All students who took part in the study were above 18 years of age so they could consent for themselves. Adherence to principles of confidentiality and anonymity was assured among the study respondents.

### 2.3 Data collection

Structured self-administered questionnaires were used as a data collection tool. The questionnaire was written in English because all respondents understood English as it is medium of instruction for their teaching and learning. Construction of the questionnaire was done by reviewing relevant literature on standard precautions for preventing TB and HIV. The study supervisors and biostatistician were consulted to review if the questions are relevant and appropriate for prevention of TB and HIV. Extra questions were added to ensure that the scope of standard precautionary measures is adequately included in the questionnaire to ensure validity. A pilot study was done on 10% of the students of the same educational level as the student of the actual study, the necessary amendments were done on the questionnaire after analysing pilot study data. It was assured that students who were part of the pilot study did not partake in the main study. The questionnaire contained closed-ended questions. These questions had a three-point Linkert scales with the following frequency responses: never, sometimes and always. Of the 144 questionnaires distributed only 107 came back. Two of those were incomplete. To guarantee anonymity, completed questionnaires (105) were returned to the class representatives in the absence of the researcher. Questionnaires were collected a week after the day of issuance.

### 2.4 Data analysis

For ease of data analysis, data was coded and captured into a spreadsheet. IBM SPSS Statistics software version 26 was used to analyse the data. The researchers also engaged the services of a qualified statistician to assist with data interpretation. Graphs, frequency tables and percentage distribution were used to present the demographics. Inferential statistics were used to analyse the data. Analysis of variables (ANOVA) and Pearson’s chi-square tests were also performed to assess correlations of the responses of the participants and their demographics. The significance level of the study was set at p < 0.05. The compliance score was reached by calculating the mean or average across the items dealing with compliance.

## 3. Results

### 3.1 Demographics results

Table 1 shows the demographic results. Among the students who participated in the study, 62.9% (n = 66) of the respondents were female, while 37.1% (n = 39) were male. All participants were between ages 20–48 years. The median age is 28 years, the mean age was 29 years and the standard deviation is 6.761. A small group of the students, 29.5% (n = 31) were married and the rest of them, 70.5% (n = 74) were single. Out of 105 students that responded, 27.6% (n = 29) of the students were doing 3^rd^ year, 54.3% (n = 57) were doing their 4^th^ year while 18.1% (n = 19) were doing their 5^th^ year. Only 3.8% (n = 4) of the students have repeated a class in their present nursing degree. Twenty-seven of the students were specialising in general nursing, others midwifery which are 32. Mental health and community had 24 and 33 of the students respectively, while 3.8% (n = 4) were doing other specialties. However, students were allowed to do more than one specialty.

**Table 1:** Respondent’s demographics.

VARIABLES	RESPONDENTS (N)	PERCENTAGE (%)
**GENDER**		
Male	39	37.1
Female	66	62.9
**AGE GROUP**		
20–24 years	40	38.1
25–29 years	17	16.2
30–34 years	21	20.0
35–39 years	20	19.0
40–44 years	3	2.9
45 and above	3	2.9
Not stated	1	1.0
**MARITAL STATUS**		
Married	31	29.5
Single	74	70.5
**LEVEL OF STUDY**		
3^rd^ year	29	27.6
4^th^ year	57	54.3
5^th^ year	19	18.1
**REPEATED YEAR**		
Yes	4	3.8
No	100	95.2
Not stated	1	1.0
**STUDY SPECIALTY**		
General Nursing	27	25.7
Midwifery	32	30.5
Mental health	24	22.9
Community Nursing	33	31.4
Other	4	3.8
Not stated	1	1.0

### 3.2 Compliance with standard precautions for prevention of TB and HIV among student nurses

**Fig 1:**
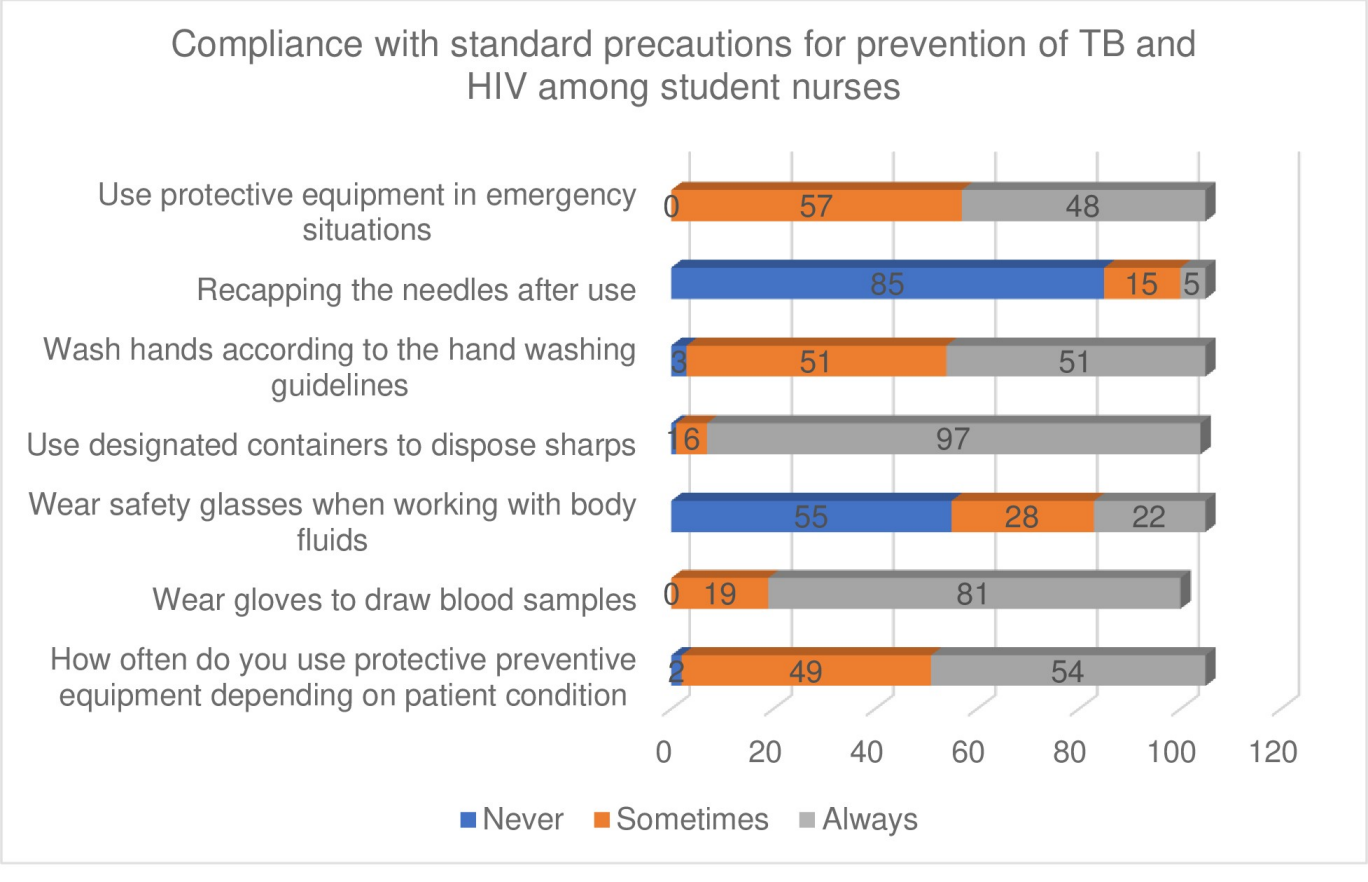
Compliance with standard precautions for prevention of TB and HIV among student nurses.

Among the various elements of standard precautions, the responses of students differed sometimes the compliance rate was low and sometimes it was high. As summarized in Fig 1, when students were asked about the use of protective preventive equipment 1.9% (n = 2) responded that they never use personal protective equipment depending on the patient’s condition while 46.7% (n = 49) said sometimes and 51.4% (n = 54) said always. Regarding wearing gloves to draw blood samples only 95.2% (n = 100) participants answered of which 18.1% (n = 19) said they sometimes wear them while most students, 77.1% (n = 81), said they wear them always. More than half of the students, 52.4% (n = 55) said they never wear safety glasses when working with body fluids while 26.7% (n = 28) sometimes wear them and only 21% (n = 22) wear them always. The student nurses were divided when it comes to hand washing as only 2.9% (n = 3) said they never wash hands according to the hand washing guidelines while 48.6% (n = 51) said sometimes and 48.6% (n = 51) said always. When it is an emergency more than half of the students, 54.3% (n = 57) admit that they sometimes use protective equipment and the other 45,7% (n = 48) use it always.

Students seem to be doing well when it comes to using designated containers to dispose sharps as only 1% (n = 1) never use designated sharp containers while 5.7% (n = 6) said sometimes and most of them most of them, 92.4% (n = 97) always use them which is good. 81% (n = 85) of the student reported to never recap the needles after use which is good but 14.3% (n = 15) do it sometimes and only 4.8% (n = 5) always recap used needles.

### 3.3 Association between demographics and compliance to standard precautions

Analysis was done to assess if there was any association between participant’s demographics and compliance to standard precautions. This study reports that there is enough evidence to show that there is association between participant’s demographics and their responses. The single questions on compliance were combined into a sub scale by computing the means across the relevant items. All scores were rounded off to at most, two decimal places, in the discussion for simple presentation and to ensure easy readability. The ANOVAs and chi square tests were done to assess association on the results on student nurses’ compliance with the demographics.

#### 3.3.1 ANOVAs’ associations

Generally, students do not comply well to standard precautions, as the mean compliance score was 2.3 the minimum being 1.7 and the maximum was 3. As seen in Table 2, male students complied more (2.31) than the female students (2.28). Students between ages 25–29 years had the highest compliance rate of 2.34, while those of ages 40–44 had the lowest compliance rate of 2.18. The compliance rate was higher on single students (2.3) as compared to married student (2.2). Fourth year students complied more with a compliance score of 2.33, followed by fifth year students at 2.27- and third-year students complied less at 2.22.

**Table 2:** Association of each of the research question to the demographics.

		N	COMPLIANCE SCALE		
			MEAN	STD. DEVIATION	P-VALUE
Gender	Male	39	2.3071	0.255	
	Female	66	2.2803	0.265	0.613
	Total	105	2.2902	0.260	
Age	20–24 years	40	2.2500	0.237	
	25–29 years	17	2.3445	0.258	
	30–34 years	21	2.3231	0.291	
	35–39 years	20	2.3000	0.285	0.757
	40–44 years	3	2.1825	0.310	
	45–49 years	3	2.2381	0.218	
	Total	104	2.2875	0.255	
Marital status	Married	31	2.2458	0.280	
	Single	74	2.3089	0.252	0.259
	Total	105	2.2902	0.260	
Level of study	3^rd^ year	29	2.2167	0.225	
	4^th^ year	57	2.3342	0.277	0.132
	5^th^ year	19	2.2707	0.242	
	Total	105	2.2902	0.260	

#### 3.3.2 Pearson’s chi squared test results

These findings were illustrated in Table 3.

**Table 3:** Association between demographics and compliance to standard precautions.

DEMOGRAPHICS	COMPLIANCE TO STANDARD PRECAUTIONS	CHI SQUARE	DEGREE OF FREEDOM	EXACT SIGNIFICANCE VALUE
Age	How often do you wash hands according to the hand washing guidelines	24.131^a^	**.014**	10
Gender	How often do you use designated containers to dispose sharps	7.545^a^	**.011**	2
Marital status	How often do you recap the needles after use	7.221^a^	**.027**	1
Study level	How often do you use protective preventive equipment depending on the patient condition	10.042^a^	**.040**	4

Cross tabulation was done on responses of compliance among student nurses and their demographics. As seen in Table 3, there was a significant relationship between compliance to hand washing and age. The exact significance value was 0.014 and the chi square test value was 24.13. There was also a correlation between gender and discarding sharps (exact significant value of 0.011; chi square test = 7.55). There was a significant relationship between recapping of used needles and marital status the exact significant value was 0.027 (chi square test = 7.22). There was also a correlation between the use of personal protective equipment (PPE) and the level of study (exact significant value was 0.40; chi square test = 10.04).

## 3.4 Discussion

Compliance to standard precautions is important to prevent the spread of TB and HIV in the clinical area. Even though student nurses are taught about its importance in class and in the clinical area, it does not mean that they will always comply. Among the participants, 51.4% reported that they always use PPE depending on the patient’s condition. The mean score for compliance was 2.29 which shows that student do not comply well. A study on knowledge and practice of universal precautions among student nurses in school of nursing revealed that 52.6% of the student nurses reported to use gloves and face mask when caring for patients [12]. However, in another study when asking student nurses on their compliance, only a very few (2.5%) said they wore protective gear/apron [13]. Gloves are also supposed to be worn by health workers when doing procedures where they anticipate contact with patients’ blood or body fluids. In this study 77.1% of the student said they always wear gloves to draw blood samples. This was similar to a study done previously where 96% of the participants said they always use gloves when they anticipate exposure to blood or bodily fluids [14]. On the contrary, in another study nurses commented that using gloves to draw blood from a patient reduces their dexterity, saying that they cannot feel the vein because “the gloves interfere” [15].

In the current study, only 21% of the students said they always wear safety glasses. This may be due to unavailability of goggles and face shields in the clinical learning environment or the lack of culture of wearing them. This is different from the result found in Saudi university where the compliance rate in wearing goggles, face shield, and apron whenever there was a possibility of a splash or splatter was 72.9% [16]. When students were asked if they used designated containers to dispose sharps in this study, 92.4% said they always did and 81% of the students said they never recap needles after use. The findings from Philippines also correspond with this current study given that 82.8% students reported that they disposed needles and blades in a sharps disposal box or receptacle after using, and almost three fourths (74.14%) said they do not recap syringe after using [17].

Washing hands has its challenges, while most people try to wash their hands often, only a few do it the right way. In this study almost half of the students 48.6% said they sometimes wash hands according to the hand washing guidelines. A previous study on hand washing gathered that 50.0% of students washed their hands 1–3 times and only 0.9% washed their hands 10 times or more [18]. In terms of the students’ duration of hand washing, the hand washing duration of 60 second or above was only done by 4.7% [18]. Compliance to using PPE in emergency situations is difficult. In this study 45.7% of the student nurses said they always used protective equipment in emergency situations. When nurses were interviewed in another study on this issue many participants described an emergency situation as a major obstacle in following precautions and participants explained how in the matter of life and death, they had to neglect their own safety and rushed to help save the patient’s life [15].

Compliance with standard precautions differed with the students’ gender, age, marital status and level of study. In this study, male students complied more than female students and the results show that there is association (Chi-square test value = 7.55; p-value = 0.009) between gender and the use of designated containers to dispose sharps. This is contrary to the result found in Northwest Ethiopia where female healthcare workers were 2.18 times more likely to be always compliant with standard precautions as compared to male HCWs [19]. Students between ages 25–29 years had the highest compliance rate while those ages 40–44 had the lowest compliance rate. There was a significant association (Chi-square test value = 24.13; p-value = 0.004) specifically on age and hand washing. This differed from a study done in Saudi Arabia where the increased in age, marital status and year of study influenced standard precautions compliance rate [20]. The compliance rate was higher on single students as compared to married student. It was worth noting that there was a significant association (Chi-square test value = 7.22; p-value = 0.003) between marital status and recapping of used needles. Fourth year students complied more to standard precautions, followed by fifth year students and third year students complied less. The difference was specifically noted in the use of PPE where an association (Chi-square test value = 10.04; p-value = 0.079) was seen in the study level and use of PPE. In a study on “Compliance with standard precautions among baccalaureate nursing students”, students at a high level of study complied more to standard precautions than students on the lower level [16]. So, their conclusion was that as the students’ exposure to the clinical environment increases, so also their compliance with standard precautions.

## 4. Conclusion

Standard precautions measures are recommended to be implemented by all student nurses to prevent the spread of infection. However, these precautions will not work if student nurses do not comply to them. Results from this study show that student nurses do not comply to standard precautions as they should. They do not wear aprons, goggles, and masks to protect themselves from blood and body fluids in the CLE. It is worth noting though that they do comply in terms of handling and disposing sharp objects after use. With this poor adherence to standard precautions, student nurses are at risk of infecting themselves and their patients during their practice in the clinical area. Adherence to the standard precautionary measures by the student nurses requires a greater investment in teaching, in-service training and practice by the students, nurse educators, clinical practice / hospital management and mentors in order to support, promote and strengthen the safety culture. Adherence to standard precautionary measures should be closely monitored to ensure total and better compliance. From these results it is recommended that clinical facilitator should always accompany student nurses in the clinical area to ensure that student nurses are supervised. The preceptors should also be exempted from other nursing duties when there are student nurses in the hospitals so that they can mentor the students.

## Supporting information

S1 Checklist Standard precautions for preventing tuberculosis and HIV: Compliance of Eswatini university student nurses. (DOCX)
